# The Organon Society, of Boston

**Published:** 1888-02

**Authors:** S. A. Kimball

**Affiliations:** Secretary


### THE ORGANON SOCIETY OF BOSTON.

The regular meeting of the Organon Society was held December 29th, 1887. There were nineteen members present.

The reading of the introduction to the *Organon* was continued.

In regard to bloodletting, Dr. Wheeler thought there was a reaction in its favor among old school physicians, and that much more of it was done now than formerly. Dr. Nichols spoke of an allopathic surgeon who bled quite frequently.

Dr. Bell related a case of glaucoma, where leeches had been applied to the temples without relief. There were terrible pains in and about the eye, worse at night and on lying down; better from warmth and from walking; had to walk most of the time. Rhus$^{cm}$ gave much relief, and some days later Rhus$^{cmm}$ cured.

In regard to the suppression of itch, Dr. Nichols related a case of primary itch cured in three months by Sulphur, internally, with no local applications but soap and water for cleanliness.

Dr. Wesselhœft related a case of a family in which all had the itch, and all were cured within a year, each by a different remedy.

It was questioned, if in a Sulphur case the external application of Sulphur would cure, but it was decided that so much of the remedy was ordinarily used in external applications that the case would be aggravated and finally suppressed.

Dr. Wesselhœft, in answer to the question, what should be told the patients when treated for such a disease, as to the length of time required for a cure, said he told them as he told his gonorrhœal or syphilitic patients, that it would take until they were cured, but that meant a cure, not a suppression. He thought it took no longer, on the average, to cure the patient homœopathically than to suppress the itch allopathically, as it often required weeks and months to suppress such cases by external applications.

Dr. Jameson related a case of morning diarrhœa cured by Aloes, and followed by an itch eruption on the hands suppressed years before.

Dr. Wesselhœft related a case, occurring in the first year of his practice, of a shoemaker with a severe asthma, with goneness in the pit of the stomach, 10–11 A. M. One dose of Sulphur$^{30}$ was given dry, and the patient returned in ten days with his asthma entirely relieved, but with an itch on his hands that had been suppressed three times before.

He also spoke of a case his father treated. An old man in East Boston, who was in very good health but was badly troubled with constipation. He had been through the usual siege with pills, etc., until he was tired, and, his constipation being no better, he had given up all treatment of that sort.

He came to the elder Dr. W. for treatment for a so-called bilious attack. When this improved he broke out with an eruption from head to foot, but during the time the eruption lasted, and it was very severe, he had a natural operation every day.

It was very strange that so slight an internal trouble should take on such a severe external expression. Dr. Bell said we were often told how much Homœopathy had done in changing old-school treatment for the better; but he did not think so. They were just as bad as ever. Their pills were smaller but worse.

In regard to the removal of tumors, Dr. Bell said that ovarian tumors usually came too late for remedial measures and must be removed, for fear that the mechanical pressure might be fatal. Cancers also were usually brought to the physician's notice when far advanced. In the early stages, no doubt, many tumors could be cured by homœopathic treatment.

Dr. Wesselhœft thought such patients should be put through a course of strict antipsoric treatment before the operation. This would prevent the return of the disease in another form.

Dr. Bell said that ovariotomy was often followed by cancer.

In regard to pleurisy with effusion, an operation was sometimes necessary.

In regard to fistula in ano, Dr. Wesselhœft spoke of the case of Theodore Parker, the members of whose family usually died of pulmonary disease in their fifty-eighth or fifty-ninth year. He had no evidence of lung trouble whatever, but had a very painful fistula. At the urgent solicitations of his family and friends, and against the strong advice of Dr. W., who warned them of the result, an operation was performed. Fourteen days after the operation he had a severe hemorrhage from the lungs, which was the beginning of the end. He soon went abroad for his health and there died.

Dr. W. related a case of *painless* fistula cured completely by Calc. phos., the lack of pain being the indication for the remedy.

In regard to operations for piles, Dr. Thurston related a case where the patient had a single pile, and was otherwise perfectly healthy. One of these hæmorrhoidal experimenters got hold of him, injected something into the pile, and the patient died in twenty-four hours from apoplexy.

Dr. Wesselhœft remarked how strange it was that Hahnemann said nothing of inherited tendencies—peculiarities transmitted from parents to children. The semen must have a tremendous dynamic force to transmit such things. He related two interesting cases. One in which the father, in his twelfth year, had a wart on his forehead; his daughter, in her twelfth year, had a wart in the same place. In the other case the father, when forty-five years of age, had a spot of eczema on his right shin; the son, in his forty-fifth year, had a similar spot on his right shin.

Adjourned to January 12th, 1888.

S. A. KIMBALL, *Secretary.*

